# Observations on the Sebacic Acid

**Published:** 1803-01-01

**Authors:** 


### *Observations on the Sebacic Acid, by* Cit. THENARS; *communicated by our Correspondent at Paris.*

THAT volatile matter, of a penetrating and even suffocating smell, which is disengaged in the distillation of fat or grease, the chemists had regarded as a particular acid, to which they gave the name of *Sebacic Acid*; Cit. Thenars however, endeavours to prove, that the true sebacic acid has been unknown till now, and is possesed of quite different characters to what is generally adopted. He proposes two methods of obtaining the true sebacic acid; the first of which, as the most simple, consists in distilling the fat by one fire, and in washing the product of the distillation with boiling water; which being afterwards filtrated, and evaporated, an acid, crystallised in form of needles, will be obtained. The second method is more complicated, though we are much surer to get by it that acid in a greater degree of purity. The water with which the product of the distillation of fat has been washed, is saturated with pot-ash; the sebat of pot-ash that is thus produced is afterwards decomposed with a solution of lead, by which a flaky precipitate of sebat of lead is produced, which being decomposed with sulphuric acid, yields, by adulcoration and evaporation, a pure sebacic acid. This acid has a slightly sourish taste, is not coloured, and melts in the manner of grease. It is much more soluble in hot than in cold water: boiling water, perfectly saturated with sebacic acid, coagulates on becoming cool. Alcohol dissolves likewise a considerable quantity of it. When it is carefully evaporated from these solutions, it may be obtained in form of very large and brilliant laminas. Sebacic acid precipitates the the acetit and nitrat of lead, the nitrat of silver, the acetit and nitrat of mercury. It forms, with pot-ash, a soluble salt of inconsiderable taste, which does not attract moisture from the atmosphere. It does not render turbid either lime water or the water of barytes and of strontianites. The preceding experiments evidently show the presence of a particular acid, produced by the distillation of fat; but the nature of this product remains to be exactly examined, and, in which circumstance, the error of chemists, with respect to the true nature of this acid, originates. On treating the product obtained by the distillation of fat with water, and on saturating this water with pot-ash, a saline mass will be obtained by evaporation; when this is dry, put it into a retort, and pour to it some diluted sulphuric acid. On distilling this mixture, an acid will be disengaged, which has all the known characters of acetous acid; the proportion of this and the sebacic acid in the products of the distillation of fat, differ according to the degree of heat that has been employed. Cit. Thenars thinks, that the pungent smell of the distilled fat may be owing to a part of this matter being decomposed, and reduced to the form of a gas, which is not acid, as it neither reddens the blue vegetable colours, nor combines itself with pot-ash. Mr. Crell, and the chemists of Dijon, have considered the sebacic acid as volatile and of a pungent smell, which error seems to have arisen from the acetous acid, which is disengaged by treating the above combination with pot-ash by sulphuric acid, and perhaps also from the muriatic acid, with which the common pot-ash is frequently impregnated, and which is also disengaged by the sulphuric acid.

